# Supplementary material for: Genetic profiling via a novel PCR-RFLP method enabled identification of four genera of anaerobic gut fungi isolated from nyala, giraffe, and zebra hosts
Source: FEMS Microbiol Lett. 2026 Oct 29;373:fnag131. doi: 10.1093/femsle/fnag131 (PMC13738085; doi:10.1093/femsle/fnag131)
Supplement: Supplementary file 1 [file fnag131_supplemental_files.zip › Supplementary_figures.docx]

**Genetic profiling via a novel PCR-RFLP method enabled identification of four genera of anaerobic gut fungi isolated from nyala, giraffe, and zebra hosts**

Supplementary information

**Figure S1.** Gas pressure and visual growth observations for initial isolates from nyala and giraffe and found discrepancies between both observations.

**Figure S2.** Growth curves of axenic fungal cultures. Cumulative fermentation gas production over time in cultures with glucose, fructose and galacturonic acid as sole carbon source, of **(A)** *N. cameroonii* isolates N2, N6, N9, N13 and **(B)** *F. austinii* isolates G2, G3, G5. Values are mean ± standard deviation (n=3).

**Figure S3.** Integration of RFLP with ITS sequence information. Visualisation of RFLP profiles predicted for currently established AGF genera as derived from in silico digestion with **(A)** DraI and **(B)** HinfI digestion. *Nf Neocallimastix frontalis; Nc N. cameroonii; Ad Aestipascuomyces dupliciliberans, Gc Ghazellomyces constrictus; Fa Feramyces austinii ; Cc Caecomyces communis; Ca Cyllamyces aberensis; As Anaeromyces spp; Oj Orpinomyces joyonii, Pr Pecaromyces ruminatium; Ja Joblinomyces apicalis; Tm Tahromyces munnarensis; Ps Piromyces* spp*.; Be Buwchfawromyces eastonii; Kr Khoyollomyces ramosus; Cs Capellomyces spp.; Liebetanzomyces polymorphus; Al Agrisomyces longus; Ap Aklioshbomyces papillarum; Tg Testudinimyces gracilis; Ad Astrotestudinimyces divisus.*

**Figure S4.** PCR-RFLP of selected isolates from giraffe. ITS rRNA region digested with **(A)** DraI or **(B)** with HinfI.
